# Effect of Metal Modification of Activated Carbon on the Hydrogen Adsorption Capacity

**DOI:** 10.3390/nano15191503

**Published:** 2025-10-01

**Authors:** Nurlan Idrissov, Nursultan Aidarbekov, Zhengisbek Kuspanov, Kydyr Askaruly, Olga Tsurtsumia, Kairat Kuterbekov, Zhassulan Zeinulla, Kenzhebatyr Bekmyrza, Asset Kabyshev, Marzhan Kubenova, Aigerim Serik

**Affiliations:** 1 Department of Materials Science, Nanotechnology and Engineering Physics, Satbayev University, Almaty 050032, Kazakhstan; kantsevich.n.v@gmail.com (N.I.); zhenis.kuspanov@gmail.com (Z.K.);; 2 Electrochemical Conversion and Energy Storage Laboratory, Institute of Nuclear Physics, Almaty 050032, Kazakhstan; 3 Faculty of Transport and Energy, L.N. Gumilyov Eurasian National University, Astana 010008, Kazakhstan; aidarbekov_nk_1@enu.kz (N.A.); bekmyrza_kzh@enu.kz (K.B.);; 4 Georgian Technical University, Tbilisi 0160, Georgia

**Keywords:** activated carbon, hydrogen storage, adsorption, magnesium, nickel

## Abstract

This study investigates the hydrogen adsorption performance of activated carbon (AC) derived from rice husks and modified with magnesium and nickel salts. Adsorption isotherms were recorded at 25 °C and 50 °C up to 80 bar, simulating practical storage conditions. The unmodified AC exhibited the highest hydrogen uptake (0.62 wt% at 25 °C), attributed to its high surface area and dominant ultramicroporosity (<0.9 nm). Modifications with Mg and Ni reduced adsorption capacity, likely due to partial pore blockage and decreased surface functionality, as confirmed by FTIR, Raman, and XRD analyses. Despite this, all samples demonstrated stable cyclic adsorption–desorption behavior and consistent isotherm profiles. Hysteresis observed in the modified samples suggests capillary condensation within mesopores. Thermodynamic analysis confirmed the exothermic nature of hydrogen adsorption. Among the modified materials, ACM10 (Mg-modified) exhibited the best performance (0.54 wt%), highlighting the importance of optimizing the metal content. The obtained results indicate that the micropore size distribution and accessible surface functionality critically govern the hydrogen storage capacity, suggesting that unmodified AC is a promising candidate for low-temperature hydrogen storage systems.

## 1. Introduction

In response to escalating environmental concerns, the transition to renewable energy sources—such as solar, wind, and hydroelectric power—has become increasingly topical and urgent [1,2,3,4]. These sources emit minimal greenhouse gases, significantly contributing to the reduction in the energy sector’s carbon footprint [5,6]. However, their dependence on external factors—such as sunlight, wind intensity, or geographic location—introduces fluctuations in power generation and causes an imbalance between supply and demand [7].

To mitigate this variability, various energy storage technological systems have been developed, including batteries, compressed-air energy storage, pumped hydro systems, flywheels, and thermal accumulators. These technologies, however, face challenges such as technical limitations, varying efficiency, safety concerns, and high initial investment costs compared to conventional systems [8,9].

A promising alternative involves converting surplus renewable energy into hydrogen for storage [10,11]. Hydrogen offers an exceptionally high gravimetric energy density—approximately 120 MJ/kg—surpassing that of gasoline by more than a factor of three [12]. However, under standard conditions (0 °C, 1 atm), gaseous hydrogen has a very low density of 0.08988 g/L [13,14]; therefore, to store sufficient amounts of hydrogen, either high pressure or cryogenic temperatures should be involved, which will eventually increase the final costs of equipment and hence the energy itself. Considering the aforementioned, improving hydrogen storage efficiency and safety is crucial for its broader application as an alternative and sustainable energy carrier [15].

Currently, physical hydrogen storage remains widespread, relying on pressure and temperature manipulation to achieve the desired phase [10]. However, the cost of these methods remains high, affecting the overall affordability of hydrogen technologies. An alternative lies in solid-state hydrogen storage, which includes both adsorption and absorption processes. According to targets established by the U.S. Department of Energy, adsorbent-based systems should ultimately reach a hydrogen storage capacity of 6.5 wt.% [16,17].

Various advanced materials—such as metal hydrides [18], zeolites, metal–organic frameworks (MOFs) [19], porous carbon materials, and activated carbon (AC)—are under investigation. Among them, AC particularly stands out due to its low cost, straightforward synthesis, and cycling stability [20,21]. It also offers a well-developed porous structure and is easily tunable, which is essential for improving adsorption properties. For instance, the incorporation of heteroatoms or metal nanoparticles can enhance hydrogen interactions and increase adsorption capacity [22,23,24].

This study aims to evaluate the effect of incorporating metal particles into AC on its hydrogen storage capacity. The AC was synthesized from rice husk using a chemical activation method. Its surface was subsequently modified with magnesium and nickel via a hydrothermal treatment using metal nitrates. Nickel is known to promote hydrogen dissociation and spillover, while magnesium provides moderate hydrogen binding energies. Their selection was motivated by their low cost and potential to enhance sorption performance, despite the possibility of partial pore blockage. Microstructural surface analysis was conducted to assess the impact of metal loading on porosity and its morphology. Hydrogen adsorption experiments were carried out at 25 °C and 50 °C under pressures ranging from 0 to 80 bar to evaluate storage performance under ambient conditions. Additionally, cyclic adsorption–desorption tests were performed to evaluate the performance of the modified materials.

## 2. Materials and Methods

### 2.1. Materials

Rice husk (sourced from the Almaty region, Kazakhstan), potassium hydroxide (KOH, 99% purity, Laborpharma, Kazakhstan), argon gas (Ar, 99.9% purity, Ikhsan Technogaz LLP), hydrogen (H_2_, 99.999% purity, Ikhsan Technogaz LLP) helium (He, 99.9% purity, Ikhsan Technogaz LLP), magnesium nitrate (Mg(NO_3_)_2_·6H_2_O, 99% purity, Laborpharma, Kazakhstan), nickel nitrate (Ni(NO_3_)_2_·6H_2_O, 99% purity, Laborpharma, Kazakhstan) were used for the experiments.

### 2.2. Synthesis of Activated Carbon

Activated carbon was synthesized from rice husk (RH) through a multi-step process involving cleaning, carbonization, and chemical activation. In the first stage, RH was thoroughly washed with cold tap water, followed by hot distilled water, and then dried at 100 °C for 10–12 h. Carbonization was performed in a vertical tubular furnace under an argon atmosphere at 500 °C, with a heating rate of approximately 5 °C/min and a 2 h holding time, followed by natural cooling.

For activation, the resulting carbon material was mixed with KOH at a 1:4 weight ratio and subjected to a second heat treatment in the same furnace at 850 °C (heating rate ~7 °C/min, holding time 90 min). Both thermal treatments were conducted under constant argon flow (450 cm^3^/min). After activation, the material was washed with a 2 mol/L HCl solution (pH 6–7), followed by multiple rinses with distilled water. The final AC product was dried at 120 °C for 10–12 h.

The experimental methodology and the rationale for the selected parameters have been discussed in detail in our previous publication [2].

### 2.3. Modification of AC

Activated carbon was modified using metal nitrates of magnesium (Mg) and nickel (Ni). Target metal loadings were set at 5 wt.%, 10 wt.%, and 20 wt.% relative to the mass of AC. The required amount of metal nitrate (Table 1) was dissolved in 50 mL of distilled water under continuous stirring with a magnetic stirrer at 40–50 °C. After complete dissolution, 2 g of pre-milled AC (ground in a planetary ball mill for 20 min) was added to the solution. The resulting mixture was stirred until a uniform suspension was formed.

The suspension was then transferred to a Teflon-lined autoclave and subjected to hydrothermal treatment at 120 °C for 12 h. Subsequently, excess moisture was removed, and the residual material was dried in a drying oven. To eliminate residual nitrates and induce activation, the resulting powder was thermally annealed under an argon atmosphere following this program: heating to 550 °C at ~7 °C/min, holding for 2 h, and allowing for natural cooling to room temperature.

The required mass of metal nitrate was calculated using the following formula:

(1) $$m_{salt}=\frac{m_{metal}*M_{salt}}{M_{metal}}$$

where M(Ni(NO_3_)_2_·6H_2_O) = 290.79 g/mol, M(Mg(NO_3_)_2_·6H_2_O) = 256.41 g/mol, MNi = 58.69, MMg = 24.31.

### 2.4. Structural Characterisation

The morphology and microstructure of the pristine and metal-modified activated carbon composites were examined using scanning electron microscopy (SEM, JSM-6510LV, JEOL, Osaka, Japan) operated at an accelerating voltage of 20 kV. Elemental distribution and composition were confirmed by energy dispersive X-ray spectroscopy (EDS). Phase composition and crystallinity were analyzed by X-ray diffraction (XRD, HZG-4A, Germany) using Cu Kα radiation (λ = 1.5418 Å) over the 2θ range of 5–70°, with a tube voltage of 40 kV and current of 40 mA. Functional groups and surface chemistry of the synthesized materials were identified by Fourier Transform Infrared Spectroscopy (FTIR, Cary 600 Series, Agilent Technologies, Santa Clara, CA, USA) equipped with an Attenuated Total Reflection (ATR) module, with a spectral range of 4000–400 cm^−1^ and resolution of 4 cm^−1^. Textural properties were characterized by N_2_ adsorption–desorption isotherms at 77 K (Micromeritics ASAP analyzer). The Brunauer–Emmett–Teller (BET) method was applied to determine the specific surface area, while the Barrett–Joyner–Halenda (BJH) model was used to calculate pore size distribution and mesopore volume. These structural parameters were directly correlated with the observed hydrogen adsorption capacities.

### 2.5. Hydrogen Adsorption Experiments

Hydrogen uptake was measured using a High-Pressure Volumetric Analyzer (HPVA-100, Micromeritics Instrument Corporation, Norcross, GA, USA). Measurements were conducted under isothermal conditions at 25 and 50 °C with equilibrium pressures ranging from 0 to 80 bar. Approximately the same mass of each sample (0.50 ± 0.01 g) was loaded into a cylindrical stainless-steel measurement cell. Prior to adsorption measurements, all samples were degassed under dynamic vacuum (<10^−3^ Torr) at 200 °C for 12 h using high-purity helium purge to remove adsorbed moisture and volatile contaminants. Instrument calibration was carried out using a non-adsorbing reference standard to account for free space volume. Adsorption–desorption cycles were repeated to confirm reproducibility.

## 3. Results and Discussion

### 3.1. Characterization

#### 3.1.1. SEM Images and Elemental Analysis of Modified Activated Carbon

**Figure 1:**
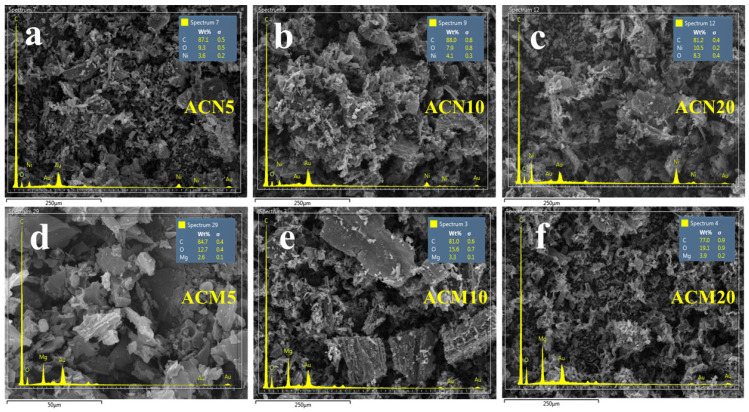
SEM images and EDX elemental mapping of activated carbon modified with different Mg and Ni loadings: (**a**–**c**) ACM5, ACM10, ACM20; (**d**–**f**) ACN5, ACN10, ACN20.

Scanning Electron Microscopy (SEM) images and energy-dispersive X-ray spectroscopy (EDS) data for activated carbon modified with magnesium (Mg) and nickel (Ni) at various loadings are shown in Figure 1. After milling, AC exhibits a loose structure with particles of varying sizes (Figure S1). Metal modification causes minor changes in surface morphology, manifested as particle agglomeration distributed across the surface. All the samples were coated with a thin layer of gold to avoid charging under the electron beam of the SEM; therefore, peaks of Au are present in the EDS spectra. EDS analysis reveals that Mg addition at 5, 10, and 20 wt.% (samples ACM5, ACM10, and ACM20, respectively) did not significantly increase Mg content on the surface, which varied only slightly between 2.6 and 3.9 wt.%, see Figure 1a–c. In contrast, Ni-modified samples (ACN5, ACN10, and ACN20) showed a progressive increase in Ni surface content from 3.6 to 8.3 wt.%, corresponding to higher metal loading (Figure 1d–f). This difference is attributed to distinct interactions with the AC surface. For example, Ni^2+^ ions interact effectively with carboxyl and hydroxyl groups on AC under acidic and neutral conditions (pH 2–7) [25,26]. Conversely, Mg^2+^ ions exhibit low solubility in these pH ranges; their optimal interaction occurs in alkaline environments (pH 8–11), facilitating stronger bonding with AC functional groups [27,28,29]. In this study, synthesis under neutral conditions likely reduced Mg fixation within the carbon structure, causing its partial loss during processing, which explains the lower surface concentration of Mg compared to Ni.

#### 3.1.2. Raman Spectroscopy Analysis

**Figure 2:**
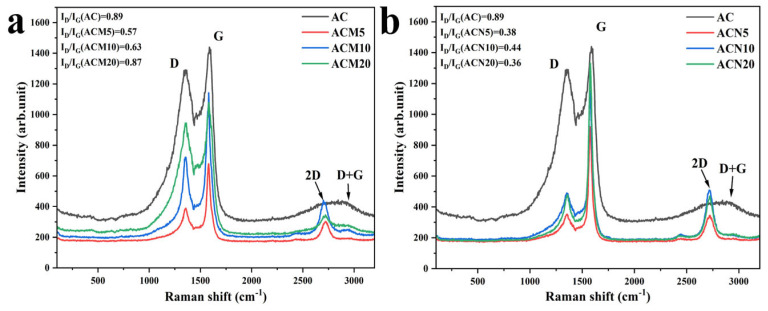
Raman spectra of the pristine activated carbon and the samples modified with metals: (**a**) magnesium and (**b**) nickel.

All synthesized samples were characterized by Raman spectroscopy in the range of 100–3200 cm^−1^ (Figure 2). The spectra of all samples exhibit two prominent characteristic peaks typical of carbon materials: the D band (~1350 cm^−1^), associated with defects and disordered structures in the carbon lattice, and the G band (~1580 cm^−1^), corresponding to the in-plane vibrations of graphitic sp^2^ carbon atoms in more ordered regions [30]. The degree of graphitization was evaluated using the intensity ratio of the D and G bands (I_D_/I_G_). For ACM5 and ACM10, a decrease in the I_D_/I_G_ ratio was observed, indicating improved structural order. However, for ACM20, the I_D_/I_G_ ratio returned to its initial value, suggesting reintroduction of structural defects at higher Mg loading. In contrast, Ni-modified samples showed a nearly twofold reduction in I_D_/I_G_, indicating a significantly higher degree of graphitization and lattice restoration. In addition to the primary bands, second-order peaks were detected, including the 2D band (~2700 cm^−1^), which is characteristic of multilayer graphene-like structures, and the D+G combination mode (~2910 cm^−1^) [31]. Pristine AC displayed a broad 2D peak, indicative of its amorphous nature (Figure 2a,b). After metal modification—especially at lower metal loadings—a reduction in 2D peak intensity was observed, suggesting partial structural ordering. Moreover, the narrowing of these second-order peaks, particularly in Ni-modified samples, further indicates enhanced crystallinity and the formation of a more homogeneous carbon structure.

#### 3.1.3. X–Ray Diffraction Analysis

**Figure 3:**
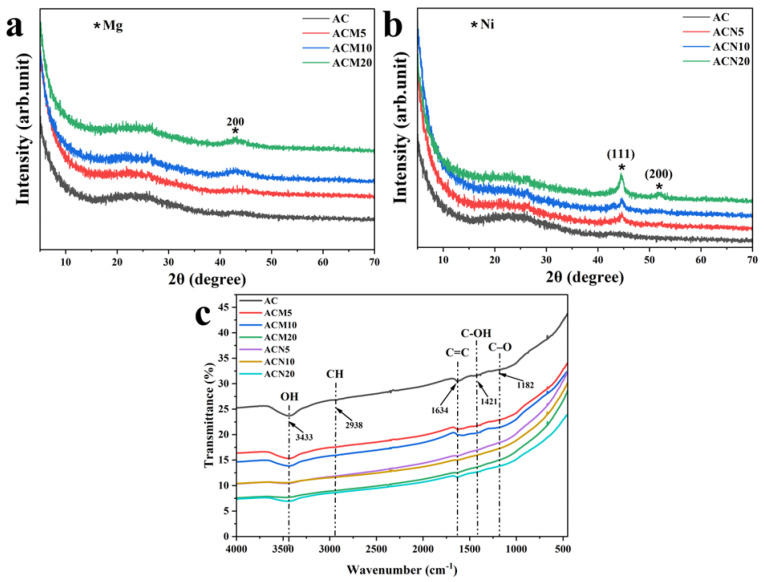
(**a**) XRD patterns of Mg–modified samples; (**b**) XRD patterns of Ni–modified samples; (**c**) FTIR spectra of pristine and modified activated carbon samples.

X–ray diffraction patterns of the samples (Figure 3a,b) confirm their predominantly amorphous nature. This is evidenced by the presence of broad, diffuse peaks in the 2θ ranges between approximately 18–33° and 40–50°, which are characteristic of graphite-like disordered carbon structures [32,33]. The pristine activated carbon exhibits a typical amorphous profile, with a broad maximum in the specified region and a weak shoulder around 43°, corresponding to the (200) plane of graphitic domains. Magnesium modification does not cause significant structural changes, although a slight increase in peak intensity near 43° is observed with increasing Mg content. Nickel–modified samples (ACN5, ACN10, ACN20) retain their amorphous nature but display notable changes in the shape and intensity of diffraction peaks. In particular, ACN10 and ACN20 show enhanced signals near 2θ ≈ 43°, suggesting the presence of Ni species and partial crystallization of the carbon matrix.

#### 3.1.4. FTIR and Textural Characterization

Fourier-transform infrared (FTIR) spectra of the pristine AC and metal-modified samples (Figure 3c) confirmed the presence of typical surface functional groups associated with carbon materials. All samples exhibited a broad absorption band near 3433 cm^−1^, corresponding to the O–H stretching vibrations of hydroxyl groups, which are present both as surface moisture and within oxygen-containing surface functionalities. The spectrum of unmodified AC also shows a weak peak at 2938 cm^−1^, attributed to sp^3^-hybridized C–H stretching vibrations [34]. The absence of this peak in modified samples suggests thermal decomposition of alkyl fragments or their replacement during interaction with metal ions. A distinct band at 1634 cm^−1^ is assigned to C=C stretching in sp^2^-hybridized aromatic domains, indicating that the aromatic character of the carbon matrix is preserved after modification [24]. Additional bands at 1421 cm^−1^ and 1182 cm^−1^ correspond to C–OH and C–O stretching vibrations, typically associated with phenolic, carboxylic, or ether groups [35]. A significant decrease in their intensity after metal modification suggests the partial removal of surface oxygen functionalities during calcination, as well as the possible formation of coordination bonds between Mg^2+^/Ni^2+^ and surface groups [36]. The structural properties of the samples were investigated using a Kubo X1000 surface area analyzer. The specific surface area was determined by the Brunauer–Emmett–Teller (BET) method, the micropore volume was calculated using t-plot analysis, and mesopore volume and size distribution were obtained via the Barrett–Joyner–Halenda (BJH) method. According to the results summarized in Table 2, pristine AC exhibited the highest specific surface area (S_BET_ = 2164 m^2^/g). A gradual decrease in surface area was observed with increasing metal content, with ACM20 and ACN20 showing significantly reduced values of 1579 and 1381 m^2^/g, respectively.

#### 3.1.5. Nitrogen Adsorption–Desorption Isotherms and Pore Structure Analysis

**Figure 4:**
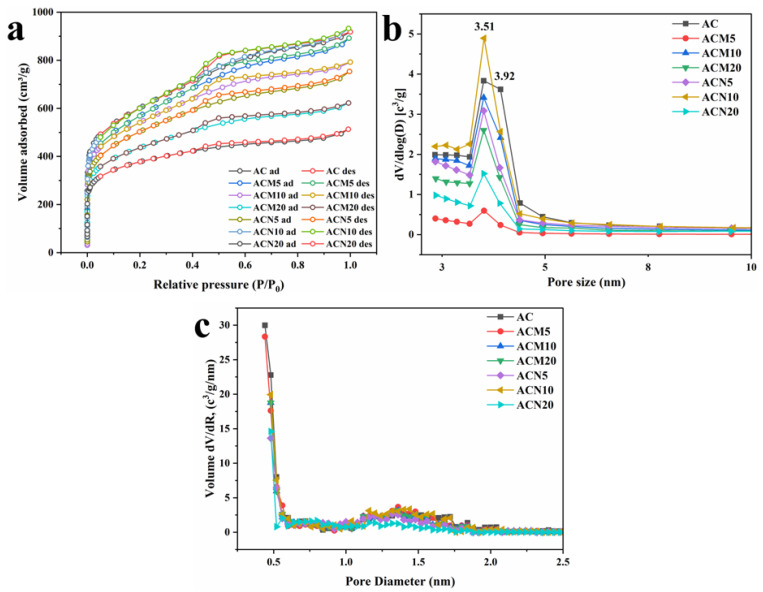
(**a**) N_2_ adsorption–desorption isotherms; (**b**) pore size distribution from BJH analysis; (**c**) micropore distribution derived from t-plot analysis.

The nitrogen adsorption–desorption isotherms of all samples (Figure 4a) are characteristic of Type IV according to the IUPAC classification, indicating the presence of both micro- and mesopores. The pronounced hysteresis loop confirms capillary condensation occurring within mesoporous structures [37]. Type IV isotherms are associated with both monolayer and multilayer adsorption: an initial monolayer forms, followed by multilayer growth and capillary filling of mesopores. The characteristic inflection point, marking the completion of monolayer formation, occurs at higher relative pressures.

Pore size distribution analysis (Figure 4b) shows that all samples exhibit a dominant pore diameter around 3.51 nm, indicating a well-developed mesoporous network. Additionally, t-plot analysis (Figure 4c) confirms the presence of micropores in all samples. For the pristine AC and ACM5, the dominant micropore size is 0.44 nm, whereas in other modified samples, it shifts slightly to 0.48 nm.

Notably, the pristine AC exhibits the highest micropore concentration, as evidenced by the maximum dV/dR value of 30 cm^3^/g/nm at approximately 0.42 nm, indicating dense micropore distribution. In contrast, metal-modified samples show significantly lower values, suggesting a partial reduction in microporosity. These results indicate that Mg and Ni salt modification leads to partial pore blockage and structural redistribution, resulting in decreased micro- and mesoporosity.

### 3.2. H_2_ Adsorption Performance

**Figure 5:**
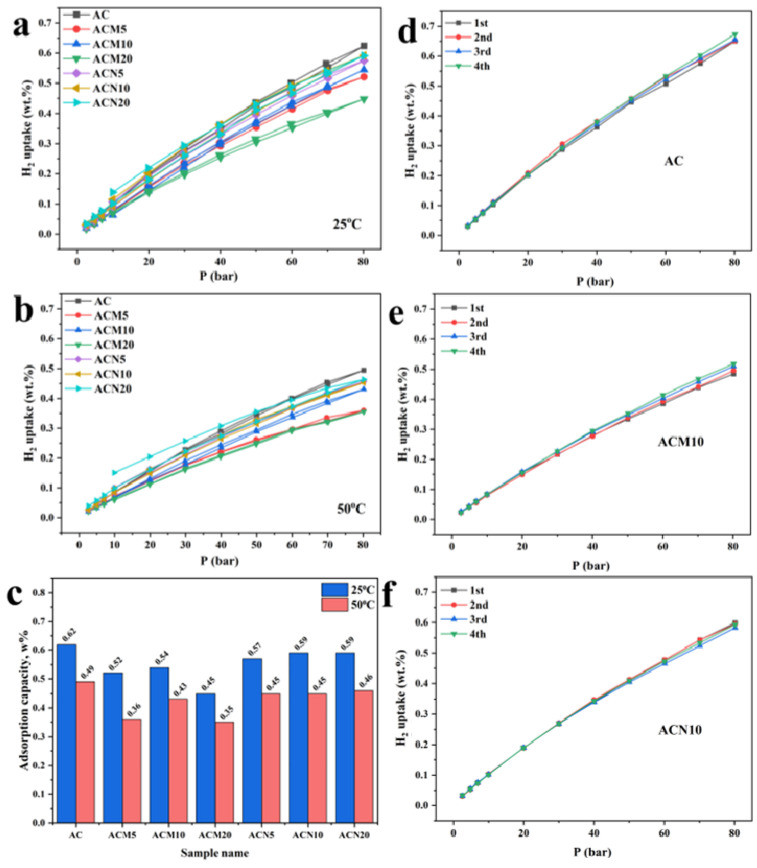
(**a**) H_2_ adsorption–desorption isotherms at 25 °C; (**b**) H_2_ adsorption–desorption isotherms at 50 °C; (**c**) hydrogen uptake at 80 bar; (**d**) cyclic adsorption–desorption performance of AC at 25 °C; (**e**) cyclic performance of ACM10 at 25 °C; (**f**) cyclic performance of ACN10 at 25 °C.

The hydrogen adsorption capacity of the synthesized samples was evaluated at 25 °C and 50 °C (Figure 5a,b), enabling assessment of performance under near-ambient conditions. As expected for physisorption, all samples exhibited a pressure-dependent increase in hydrogen uptake. However, while the absolute uptake rose with increasing pressure, the relative differences between pristine and metal-modified samples remained consistent, with unmodified AC showing the highest capacity. The isotherms maintained a nearly linear trend even at high pressures, suggesting that pore saturation was not reached. The presence of distinct hysteresis loops in the isotherms of metal-modified samples indicates capillary condensation in mesopores, which is consistent with nitrogen adsorption results [37]. Hydrogen uptake was higher at 25 °C than at 50 °C for all samples, which reflects the exothermic nature of the process, where higher temperatures reduce sorption efficiency. The highest uptake was observed for unmodified AC, reaching 0.62 wt.% at 25 °C and 80 bar. Metal salt modification with Mg and Ni did not enhance hydrogen capacity; instead, a decrease was observed compared to the pristine material. This decline can be attributed to partial pore blockage by metal particles and structural rearrangements within the carbon matrix, resulting in a decrease in the specific surface area. This interpretation is supported by XRD, Raman, and FTIR analyses, which reveal a decrease in the intensity of bands associated with oxygen-containing functional groups. Such modifications reduce the availability of surface-active sites for hydrogen adsorption. Previous studies have shown that micropores, particularly those smaller than 0.9 nm, play a crucial role in hydrogen storage [38,39]. These pore sizes enable stronger interactions with hydrogen molecules, contributing to higher sorption capacities. The pristine AC demonstrated the highest micropore volume, which accounts for its superior hydrogen adsorption performance. Figure 5c summarizes the hydrogen uptake values across all samples. Among the Mg-modified materials, ACM10 exhibited the highest capacity. Further increases in Mg content led to a decline in performance, highlighting the importance of optimizing metal loading. In contrast, Ni-modified samples did not show a clear correlation between metal content and sorption performance. Increasing Ni concentration had minimal impact on hydrogen uptake, and this trend remained consistent at both 25 °C and 50 °C.

One of the key indicators of adsorbent performance is its sorption stability under repeated cycling. Figure 5d–f display the results of four consecutive hydrogen adsorption–desorption cycles for AC, ACM10, and ACN10, respectively. All three materials demonstrated satisfactory stability, maintaining hydrogen retention capacity throughout the tests. Notably, AC and ACM10 showed a slight increase in sorption capacity—from 0.65 to 0.67 wt% and from 0.48 to 0.51 wt%, respectively—while ACN10 exhibited a minor decline. This may be attributed to pressure-induced structural changes, where high pressure could either activate previously inaccessible pores or partially damage the structure, generating new surface-active sites.

The observed trends are in line with first-principles studies, which show that Ni can create active dissociation sites and Mg can polarize H_2_, enhancing low-pressure uptake. However, these benefits are only effective when microporosity is preserved; excessive metal loading results in pore blockage and reduced sorption capacity [40,41].

The thermodynamic parameters of hydrogen adsorption for all samples are summarised in Table 3, assuming a constant adsorption of approximately 0.25 wt% at 25 °C. The enthalpy of adsorption (ΔH_ads_) was calculated using the Clausius-Clapeyron equation as described by Jarosław Serafin et al. [42]. The calculation formulas are provided in the Supplementary Information, Section “Thermodynamic Calculations”. All samples exhibited negative values of ΔH_ads_ ranging from 11.62 to −11.88 kJ/mol, confirming the exothermic nature and physical mechanism of hydrogen absorption. The Gibbs free energy of adsorption (ΔG_ads_) determined from the equilibrium pressure at 25 °C was approximately −0.03 kJ/mol, indicating spontaneous adsorption. The entropy of adsorption (ΔS_ads_), calculated from the relationship ΔS_ads_ = (ΔH_ads_ − ΔG_ads_)/T, was in the range of 39.38–40.59.3 J/(mol·K), which corresponds to an increase in disorder during adsorption and is characteristic of physisorption in porous materials [43].

## 4. Conclusions

In this study, a comprehensive investigation was conducted to assess the effect of magnesium and nickel salt modifications on the hydrogen storage capacity of activated carbon derived from rice husk. Although metal doping is a widely adopted strategy to enhance the performance of carbon-based adsorbents, our results revealed no significant improvement in sorption capacity. In contrast, a reduction in performance was observed, attributed to a decrease in specific surface area (confirmed by BET analysis) and a lower abundance of oxygen-containing functional groups (identified via FTIR spectroscopy), likely caused by partial pore blockage due to metal deposition.

The unmodified sample (AC) exhibited the highest hydrogen uptake of 0.62 wt.%. Among the modified materials, ACM10 showed the best performance with a capacity of 0.54 wt.%, while Ni-modified samples (ACN10 and ACN20) demonstrated limited enhancement. Thermodynamic analysis confirmed the exothermic nature of hydrogen adsorption, with enthalpy values ranging from −11.62 to −11.88 kJ/mol, which explains the observed decrease in capacity at elevated temperatures. Cyclic tests also demonstrated stable adsorption performance over multiple cycles.

These findings emphasize that maintaining microporosity—particularly pores <0.9 nm—is critical for achieving high hydrogen storage capacities, while excessive metal loading can hinder adsorption. Importantly, the results provide practical insights into industries: they show that simple salt impregnation does not necessarily improve storage performance, and that optimization should focus on preserving pore structure while selectively introducing active sites. This guidance is valuable for companies developing hydrogen storage systems, as it helps avoid ineffective modification routes and highlights strategies that can make biomass-derived carbons competitive in future energy technologies.

## Figures and Tables

**Table 1:** Target metal loadings and corresponding nitrate salt masses for Mg- and Ni-modified activated carbon samples.

Metal Loading	Mass of Mg (g)	Mass of Mg(NO_3_)_2_·6H_2_O (g)	Mass of Ni (g)	Mass of Ni(NO_3_)_2_·6H_2_O (g)
5%	0.1	1.05	0.1	0.5
10%	0.2	2.1	0.2	0.99
20%	0.4	4.2	0.4	1.98

**Table 2:** Textural parameters of pristine and metal-modified activated carbon samples: BET surface area, micropore volume, and mesopore volume.

Sample	BET Surface Area (m^2^/g)	Langmuir Surface Area (m^2^/g)	V_T_ (P/P0 = 0.99) (cm^3^/g)	V_μ_ (cm^3^/g)	V_m_ (cm^3^/g)	V _μ/_ V_T_	V_m_/V_T_	Dp (nm)
AC	2164	2245	1.43	0.22	0.93	0.16	0.65	3.46
ACM5	2034	2077	1.38	0.15	0.94	0.11	0.68	3.44
ACM10	1935	2005	1.23	0.18	0.81	0.15	0.66	3.28
ACM20	1579	1635	0.97	0.16	0.57	0.16	0.59	3.52
ACN5	1809	1859	1.17	0.13	0.72	0.11	0.61	3.36
ACN10	2147	2406	1.44	0.16	1.03	0.11	0.71	3.52
ACN20	1381	1450	0.79	0.16	1.86	0.2	0.5	3.72

**Table 3:** Thermodynamic parameters of hydrogen adsorption at 25 °C for all samples: adsorption.

Sample	ΔH_ads_ (kJ/mol)	ΔG_ads_ (kJ/mol)	ΔS_ads_ (J/K·mol)
AC	−11.81	−0.03	40.03
ACM5	−11.77	−0.03	39.9
ACM10	−11.88	−0.03	40.59
ACM20	−11.84	−0.03	40.15
ACN5	−11.66	−0.03	39.53
ACN10	−11.64	−0.03	39.46
ACN20	−11.62	−0.03	39.38

enthalpy (ΔH_ads_), Gibbs free energy (ΔG_ads_), and entropy (ΔS_ads_).

## Data Availability

Data are contained within the article.

## Supplementary Materials

The following supporting information can be downloaded at: https://www.mdpi.com/article/10.3390/nano15191503/s1, Figure S1: SEM images: (a) pristine activated carbon (AC); (b,c) AC after milling, shown at different magnifications.

## Nomenclature

AC	activated carbon;
ACM5/ACM10/ACM20	activated carbon modified with Mg at loads of 5/10/20 wt%;
ACN5/ACN10/ACN20	activated carbon modified with Ni at loads of 5/10/20 wt%;
S_BET_	BET specific surface area (m^2^/g);
V_μ_	micropore volume (cm^3^/g);
V_m_	mesopore volume (cm^3^/g);
q	adsorption capacity (wt%);
HPVA	High Pressure Volumetric Analyzer;
ΔH_ads_	adsorption enthalpy (kJ/mol);
ΔG_ads_	Gibbs free energy of adsorption (kJ/mol);
ΔS_ads_	entropy of adsorption (kJ/mol);
I_D_/I_G_	ratio of intensities of D- and G-peaks (Raman).
