# Supplementary material for: Effect of Metal Modification of Activated Carbon on the Hydrogen Adsorption Capacity
Source: Nanomaterials (Basel). 2025 Oct 1;15(19):1503. doi: 10.3390/nano15191503 (PMC12526254; doi:10.3390/nano15191503)
Supplement: Supplementary file 1 [file nanomaterials-15-01503-s001.zip › nanomaterials-3860297-supplementary.pdf]

Supplementary information

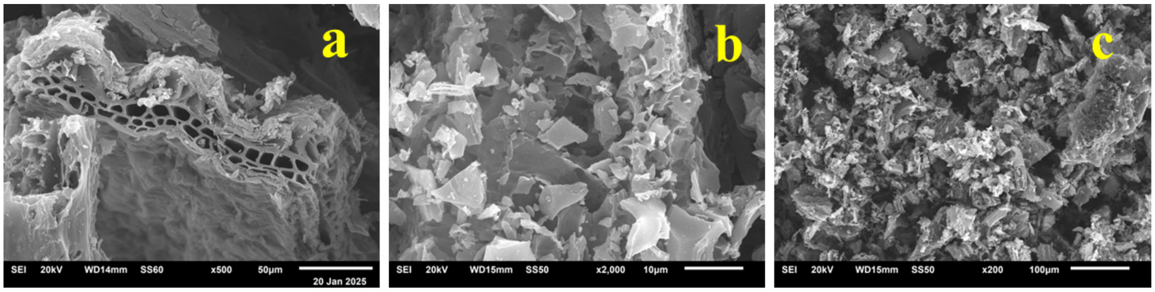

Figure S1: SEM images: (a) pristine activated carbon (AC); (b, c) AC after milling, shown at different magnifications.

Thermodynamic calculations

1) Method to obtain ΔH_{ads} (isosteric enthalpy of adsorption)

We applied the fixed-coverage Clausius–Clapeyron approach. For several coverages q_i (mmol/g), the equilibrium pressures P(T) were read from H_2 isotherms measured at different temperatures. Linear fits of lnP versus 1/T give the slope m, from which:

$q_{st}(q_i) = -R\ (\partial \ln P/\partial(1/T))_{(q_i)}$, $\Delta H_{ads}(q_i) \equiv -q_{st}(q_i)$

with R = 8.314 J/mol·K. We report ΔH_ads as negative for exothermic adsorption (its magnitude equals the isosteric heat). For each sample we provide regression plots, slopes, R^2, and 95% confidence intervals.

2) Method to obtain ΔG_{ads} and ΔS_{ads}

In the low-pressure Henry region, the isotherm is $q = K_H P$ (with q in mmol/g, P in bar). We determine K_H by linear regression of the initial isotherm. To render a dimensionless equilibrium constant, we normalize to the standard pressure P° = 1 bar: $K° = K_H P°$. Then:

$$\Delta G\_ads(T) = -RT \ln K°, \quad \Delta S_{ads}(T) = (\Delta H_{ads}(T) - \Delta G_{ads}(T)) / T.$$
